# Unsupervised registration of 3D knee implant components to biplanar X-ray images

**DOI:** 10.1186/s12880-023-01048-9

**Published:** 2023-09-18

**Authors:** Dac Cong Tai Nguyen, Said Benameur, Max Mignotte, Frédéric Lavoie

**Affiliations:** 1 Département d’Informatique et de Recherche Opérationnelle (DIRO), Université de Montréal, Montréal, Québec Canada; 2 Eiffel Medtech Inc., Montréal, Québec Canada; 3 Orthopedic Surgery Department, Centre Hospitalier de l’Université de Montréal (CHUM), Montréal, Québec Canada

**Keywords:** 3D/2D registration, X-ray images, Knee implant components, Orthopaedic implants

## Abstract

**Background:**

Registration of three-dimensional (3D) knee implant components to radiographic images provides the 3D position of the implants which aids to analyze the component alignment after total knee arthroplasty.

**Methods:**

We present an automatic 3D to two-dimensional (2D) registration using biplanar radiographic images based on a hybrid similarity measure integrating region and edge-based information. More precisely, this measure is herein defined as a weighted combination of an edge potential field-based similarity, which represents the relation between the external contours of the component projections and an edge potential field estimated on the two radiographic images, and an object specificity property, which is based on the distinction of the region-label inside and outside of the object.

**Results:**

The accuracy of our 3D/2D registration algorithm was assessed on a sample of 64 components (32 femoral components and 32 tibial components). In our tests, we obtained an average of the root mean square error (RMSE) of 0.18 mm, which is significantly lower than that of both single similarity methods, supporting our hypothesis of better stability and accuracy with the proposed approach.

**Conclusion:**

Our method, which provides six accurate registration parameters (three rotations and three translations) without requiring any fiducial markers, makes it possible to perform the important analyses on the rotational alignment of the femoral and tibial components on a large number of cases. In addition, this method can be extended to register other implants or bones.

## Background

Total knee arthroplasty (TKA) is an orthopaedic surgical procedure where the damaged articular surfaces of the knee joint are replaced with artificial implants. The implant consists of two metallic components that replace the bearing surfaces on the tibial plateau and femoral condyles, separated by a high molecular weight polyethylene insert. The component alignment after TKA has been proved as a significant factor in determining knee kinematics [1, 2], patellar tracking, and long-term clinical outcome [3, 4]. This alignment is currently evaluated in 2D X-ray images [5, 6]. However, position of the X-ray source, orientation of the subject’s pelvis and lower extremity may have an effect on measurements obtained from 2D radiographs. A 3D analyses of component positions after TKA will possibly not only to increase the accuracy of measurements, but also to lead to new works on TKA, or to improve implant designs which increase their life span because abnormal knee kinematics might cause premature failure of the implant [7, 8].

A 3D lower extremity alignment assessment system has been created to evaluate 3D alignment by manually matching 3D bone and component projections with frontal and oblique X-ray images of the entire lower extremity [4, 9]. But this system is time-consuming and has low accuracy of position estimation [10]. 3D alignment information after TKA can also be obtained from magnetic resonance imaging (MRI) and computed tomography (CT) scan [11, 12] but is costly and involves significant radiation exposure in the case of CT scans. If serial follow-up evaluations are needed, the cost and the issue of radiation will greatly increase after sequential MRI or CT scans. Another approach based on Roentgen stereophotogrammetry has been developed [13]. Roentgen stereophotogrammetric analysis (RSA) is a highly accurate technique for 3D micromotion evaluation of orthopaedic implants but it is limited by the need to surgically insert numerous tantalum beads into the bones with a special instrument.

In our case, a 3D/2D registration based method seems to be an adequate and suitable solution. 3D/2D registration methods have been used in many medical fields, mostly in image-guided therapy, such as cancer diagnosis and therapy [14, 15], radiosurgery [16], interventional radiology [17], and a variety of therapies in surgery [18–20]. In this context, these techniques align 3D implant components to 2D X-ray images to determine their 3D information (positions and orientations). These registration approaches can be classified into two groups based on the number of images used. Most of them use fluoroscopic images for 2D X-ray image.

**Fig. 1:**
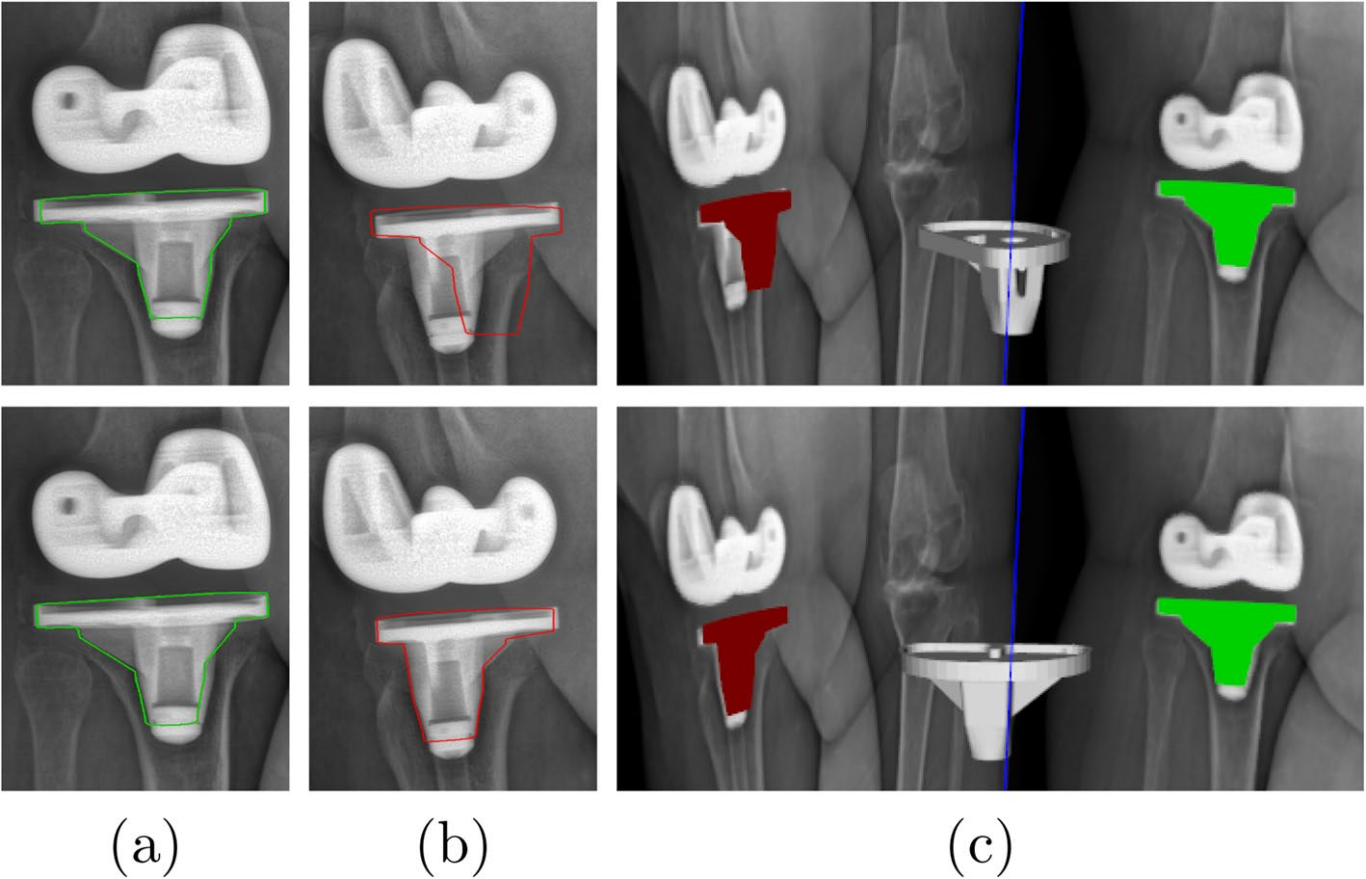
Example of tibial component projection on two X-ray images. From top to bottom : symmetrical and true pose. **a**, **b** component external contours projected on 135-degree image and 45-degree image, and (**c**) 3D view of component projections

The first group uses a single X-ray image and biplanar X-ray images are used in the second group. Previous approaches used a direct image to image similarity measure [21] or contour-based 3D/2D registration [22, 23] to estimate the pose of knee implant. Such technique can provide clinically sufficient accuracy only for five degrees of freedom (DOF) (three rotations and two translations parallel to image), as the DOF related to the translation perpendicular to image (depth position) is quite challenging. Yamazaki et al. [22] improved the depth position estimation by optimizing it independently of the five other DOFs, using an approximate evaluation curve of depth position prepared after initial registration. Although depth position was improved, it wasn’t judged to be sufficiently accurate. The RMSE, average errors and standard deviation of depth position in this technique were ten times higher than those of two other translations. Another approach to increase the accuracy of depth position estimation was based on the fluoroscopic imaging property that the closer the object is to the source, the larger the image at the image intensifier is produced. Hossain et al. [24] determined the scale change due to the depth translation by using a calibration box to estimate the depth position. This approach gave accurate results but required an extra step to compute the scale change in the depth translation of the fluoroscopy unit. The second limitation is due to the high degree of shape symmetry of the components, particularly the tibial component. A symmetrical pose might be obtained instead of the true one because the representation of both solutions on the X-ray image is very similar (see Fig. 1). In [23], the authors try to solve this problem by simultaneously estimating two symmetrical poses, but the algorithm still might not converge toward the true pose. By using biplanar 2D images, these methods can measure all six DOFs with a sufficient accuracy (see Fig. 1) and avoid the symmetrical issue. Kim et al. [25] optimized the normalized correlation coefficient (NCC) between the dual X-ray images and two corresponding virtually projected component images to obtain six DOFs. George et al. have shown that using biplanar images provides a highly accurate estimation of six DOFs [26]. However, biplanar fluoroscopic images at the same exact position are not easy to obtain due to the moving pictures and the complexity of the system. In addition, they involve a potential risk of radiation to the patient. Radiography is an adequate choice as it is the lowest in term of cost, complexity, and risk of radiation compared to fluoroscopy, MRI, or CT scan.

In this paper, we propose a simple method to align the components of a 3D knee implant to biplanar oblique X-ray images. This method does not require fiducial markers and/or intraoperative X-ray image segmentation. It uses a hybrid (relying on both region and edge-based information) similarity measure both combining the contour similarity between the external contours of the component projections and an edge potential field estimated on the two radiographic images [27] and a region label similarity measure term (which is based on the distinction of the region label inside and outside of the object [28]). Then a stochastic Exploration Selection (ES) algorithm is used to estimate the six DOFs of implant position.

## Proposed approach

### Image pre-processing

#### Contour detection

**Fig. 2:**
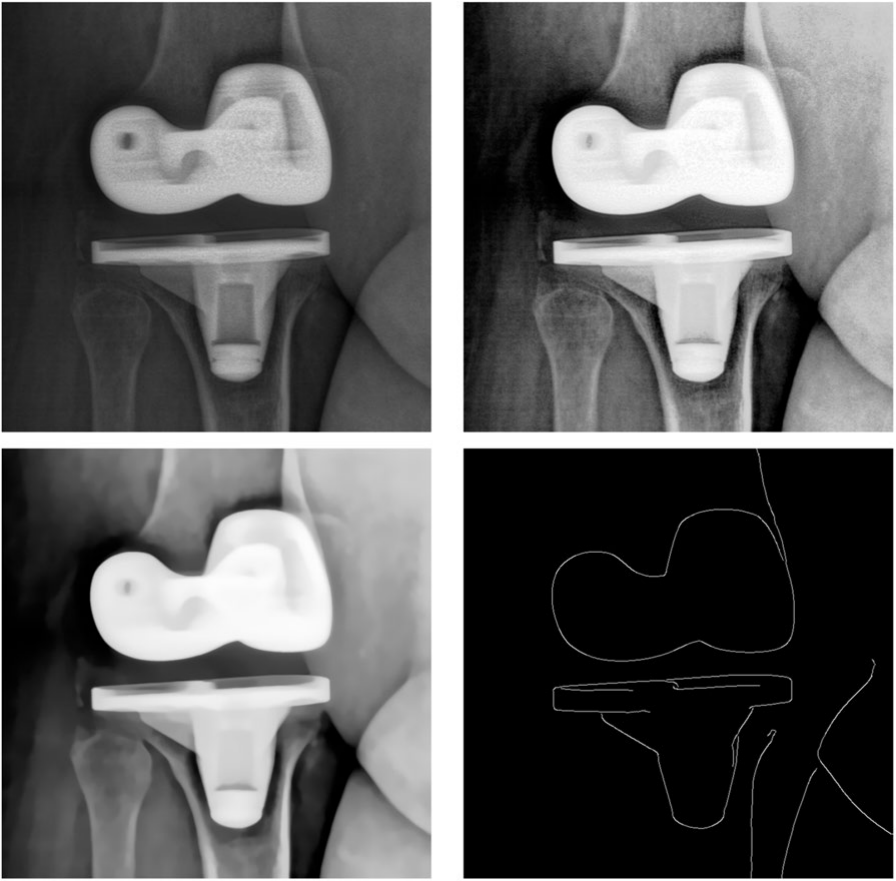
Example of contour-based pre-processing step. From left to right, first row : input X-ray image, histogram equalization image enhancement, and second row : denoised image, pre-processed image

Due to the metallic material, the implant components appear much whiter than the neighboring bones and soft tissues. A pre-processing process is performed on each image to enhance the visibility of the component contours which, in fact, constitute the most important and reliable low-level visual cue in each radiographic image. First, a histogram equalization technique increases the global contrast. Second, a median filter and non-local means denoising [29] algorithm are used to remove the noise from the images. The non-local means denoising method replaces a pixel with a weighted average of pixels having a similar neighborhood. More precisely, for each pixel, it first searches in a large search window (centered on the pixel to be denoised) for all the neighborhoods of pixels that most closely resemble (with a least squares (LSQ) similarity measure) the neighborhood of the pixel to be filtered. Then, a weighted average (based on the previous LSQ similarity measure) of all these central pixels (of all these neighborhoods) allows to estimate the denoised greyscale value of the pixel. Finally, the edges are detected by using a Canny edge filter [30] (see Fig. 2).

#### Label detection

The next part of the pre-processing step is the region-label extraction. By using the Simple Linear Iterative Clustering (SLIC) algorithm [31], the input images are segmented into *K* labels (*i.e.* superpixels). This algorithm is actually simple and has a low computational cost. SLIC performs a clustering of pixels in a five-dimensional (5D) space based on their color similarity and proximity in the image. The 5D space is defined by the *L*, *a*, *b* values of the CIELAB color space and *x*, *y* coordinates of the pixels. Due to the fact that the distance between two colors in the CIELAB space is different from the spatial distance in the xy plane, it is not possible to simply use the Euclidean distance. In order to cluster pixels in this 5D space, a new distance measure based on Euclidean distance, with normalization of the spatial distances, was introduced:

1 $$\begin{aligned} d_{Lab}= & {} \sqrt{(L_{u}-L_{v})^2 + (a_{u}-a_{v})^2 + (b_{u}-b_{v})^2}\end{aligned}$$

2 $$\begin{aligned} d_{xy}= & {} \sqrt{(x_{u}-x_{v})^2 + (y_{u}-y_{v})^2}\end{aligned}$$

3 $$\begin{aligned} D=\ & {} d_{Lab} + \frac{m}{S}d_{xy} \end{aligned}$$

where *D* is the sum of the *Lab* distance and the *xy* *plane* distance normalized by the grid interval $S=\sqrt{\frac{N}{K}}$, *N* is the number of pixels in image and *m* is a variable to controlling the compactness of a superpixel.

**Fig. 3:**
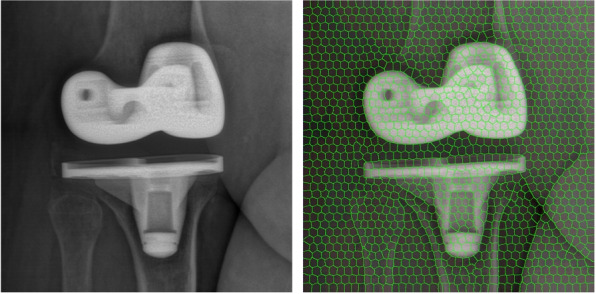
Example of label-based pre-processing step : input X-ray image (left) and pre-processed image (right)

The algorithm begins by initializing *K* cluster centers. Each pixel in the image is associated with the nearest cluster center whose search area overlaps this pixel. After all the pixels are associated, new centers are computed as the average 5D vector of all the pixels belonging to the cluster. The assignment of each pixel to the nearest cluster center and the re-computation of the new cluster center process are iteratively repeated until convergence. At the end of this process, a few remaining pixels are enforced to connect to the largest neighboring cluster (see Fig. 3).

### 3D/2D registration procedure

Once the edges and the region-based labels are extracted from the image by our pre-processing step (see “Image pre-processing” section), a rigid registration is performed to align the component to biplanar images. To this end, we combine the object specificity property [28] and the similarity between the external contours of the component projections and an edge potential field estimated on the two radiographic images.

#### Similarity measurement

##### Edge potential field-based similarity

**Fig. 4:**
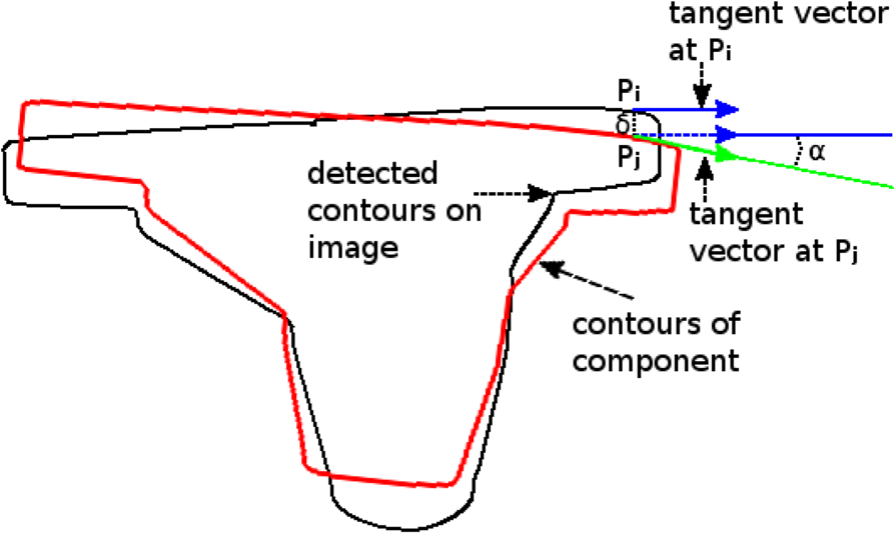
Directional component used in Eq. (5)

An edge potential field-based similarity measure is defined in order to evaluate the concordance or the similarity degree between the external contours of the component projections on biplanar X-ray images and an edge potential field, estimated from the previously detected edges of the two views. Concretely, this edge potential field attracts the component and aligns it to the edge from the input image by giving a similarity measure all the greater as the edges of the projected contours of the component coincide well with the edges existing in the two views. The edge potential field $\Phi$ of each view is computed on the pre-processed image and is defined (as in [27]) as:

4 $$\begin{aligned} \Phi (x,y) = \exp \left(-\rho \sqrt{\delta ^2_{x}+\delta ^2_{y}}\right) \end{aligned}$$

where $(\delta _{x},\delta _{y})$ is the displacement to the nearest edge point in the image, and $\rho$ is a smoothing factor which controls the degree of smoothness of the potential field. Finally, a directional component is added to produce a cost function measuring the correspondence between the projected contours of the component and the edges in the two views:

5 $$\begin{aligned} E= & {} -\frac{1}{n_{1}}\sum _{n_{1}}(\Phi _{1}(x,y) | \cos (\alpha _{1}(x,y))|)\nonumber \\{} & {} - \frac{1}{n_{2}}\sum _{n_{2}}(\Phi _{2}(x,y) | \cos (\alpha _{2}(x,y))|) \end{aligned}$$

where $\alpha (x,y)$ is the angle between the tangent direction of the external contours at (*x*, *y*) and the tangent of the nearest edge, and $n_{1},n_{2}$ are the number of pixels on external contours of the component of each view (see Fig. 4).

##### Object specificity property

Object specificity property is based on the following hypothesis : *the labels inside and outside the object are distinct*. This property is fulfilled (thus involving a minimal cost or error function) whenever the labels inside an object are specific to that object, *i.e.* the labels inside an object do not occur outside that object and vice versa. It is defined by:

6 $$\begin{aligned} \vartheta = \sum _{h=1}^{K}p_{h}|\{s:z_{s}=l_{h},s\notin c^{int}\}| \end{aligned}$$

where $\Lambda =\{l_{1},...,l_{K}\}$ is the set of *K* labels in image, $z_{s}\in \Lambda$, $p_{h}$ is the proportion of the pixels with label $l_{h}$ in the interior $c^{int}$ of component, and the last factor is the number of pixels belonging to label $l_{h}$ in the exterior of component. To reduce execution time, the last factor can be written as $|\{s:z_{s}=l_{h}\}| - |\{s:z_{s}=l_{h},s\in c^{int}\}|$, so $|\{s:z_{s}=l_{h}\}|$ can be precomputed, only $p_{h}$ is computed dynamically. By normalizing this property, we obtain a cost (or error) function (to be minimized):

7 $$\begin{aligned} V = \frac{\vartheta _{1}}{N_{1}} + \frac{\vartheta _{2}}{N_{2}} \end{aligned}$$

where $N_{1},N_{2}$ are the number of pixels of component related to each view.

#### Six registration parameters optimization

Finally, our registration approach is formulated as the minimization of the following cost function:

8 $$\begin{aligned} C(\theta ) = E(\theta ) + \beta V(\theta ) \end{aligned}$$

where $\theta$ is a set of six registration parameters and $\beta$ is a weighting factor between respectively the edge-based and region-based energy terms of our energy-based registration model. In order to minimize this complex non-convex cost function, we resort to ES algorithm, a stochastic and efficient optimization algorithm, already proposed in [32] and especially well-suited for this type of function to be optimized.

In fact, the ES algorithm belongs to the class of evolutionary algorithms. This class of algorithm inspires the natural evolution to solve hard problems. Suppose that a problem is a natural environment which encompasses a population of individuals. Each individual represents a possible solution to the problem. A fitness function is used to measure the degree of adaptation of each individual (*i.e.* potential solution) to its environment (*i.e.* problem). Like evolution in nature, these algorithms produce progressively better solution to the problem. This class of algorithm has been successfully used in medical imaging [33–35] or for detection and accurate localization of shapes in traditional images [28]. More formally, the ES algorithm can be described in two steps: exploration and selection steps (more details are given in [32]). The first step implicates a probabilistic operator to attempt a random search on a graph of each individual which is considered as a potential solution. And the second step creates interaction and selection between individuals. This process is run until a stopping criterion has been met (see Algorithm 1).

**Figure:**
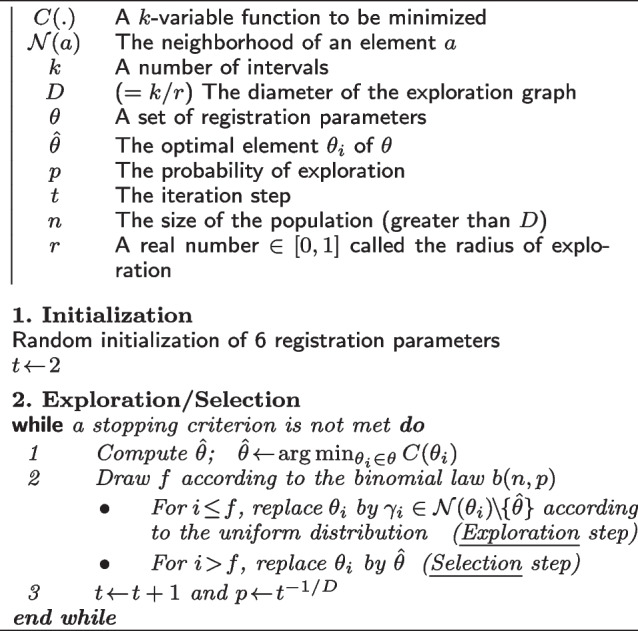
**Algorithm 1** ES Algorithm

## Experimental Results

**Fig. 5:**
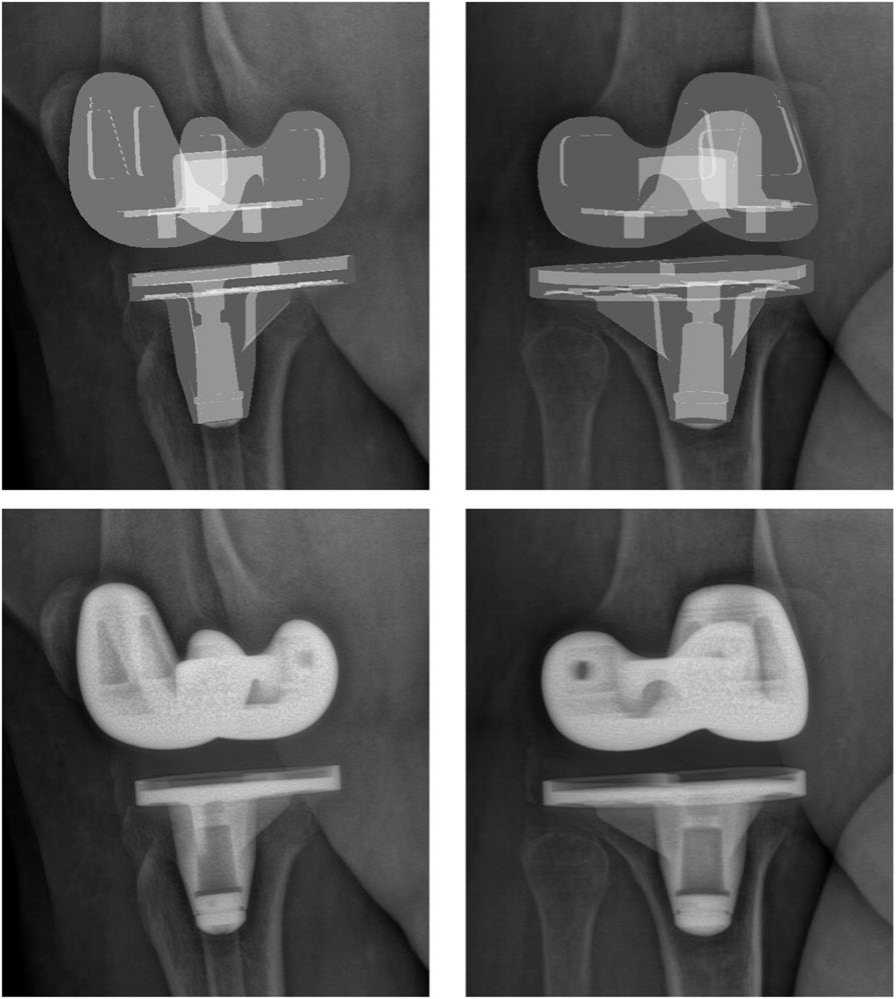
Example of biplanar ground truth images (top) and biplanar real radiographic images (bottom)

To evaluate the accuracy of our 3D/2D registration algorithm, ground truth models were created. A ground truth model is the rendering of the 3D implant components on a real X-ray image (see Fig. 5). The ground truth image retains the properties of the X-ray imaging such as variety of imaging noise originated from several components of the system (X-ray source, CCD camera, controller circuits, etc.), and the patient bone structures overlap with other bones or dense soft tissues (cartilage, meniscus, and fascia). A random transformation (rotation and translation) was applied to the ground truth 3D components. Then we compared the registered transformed components with the ground truth components. This comparison was evaluated on 64 randomly transformed components (32 femoral and 32 tibial components). In this comparison, we also performed the registrations with different similarity measurements. Afterward, these results were analyzed by using a statistical Z-test^1^ between hybrid similarity and single similarity approaches where the null hypothesis was that there is no difference between two approaches, and a statistical significance threshold set at *p* $\le$ 0.001. Then the *p*-value was calculated.

**Fig. 6:**
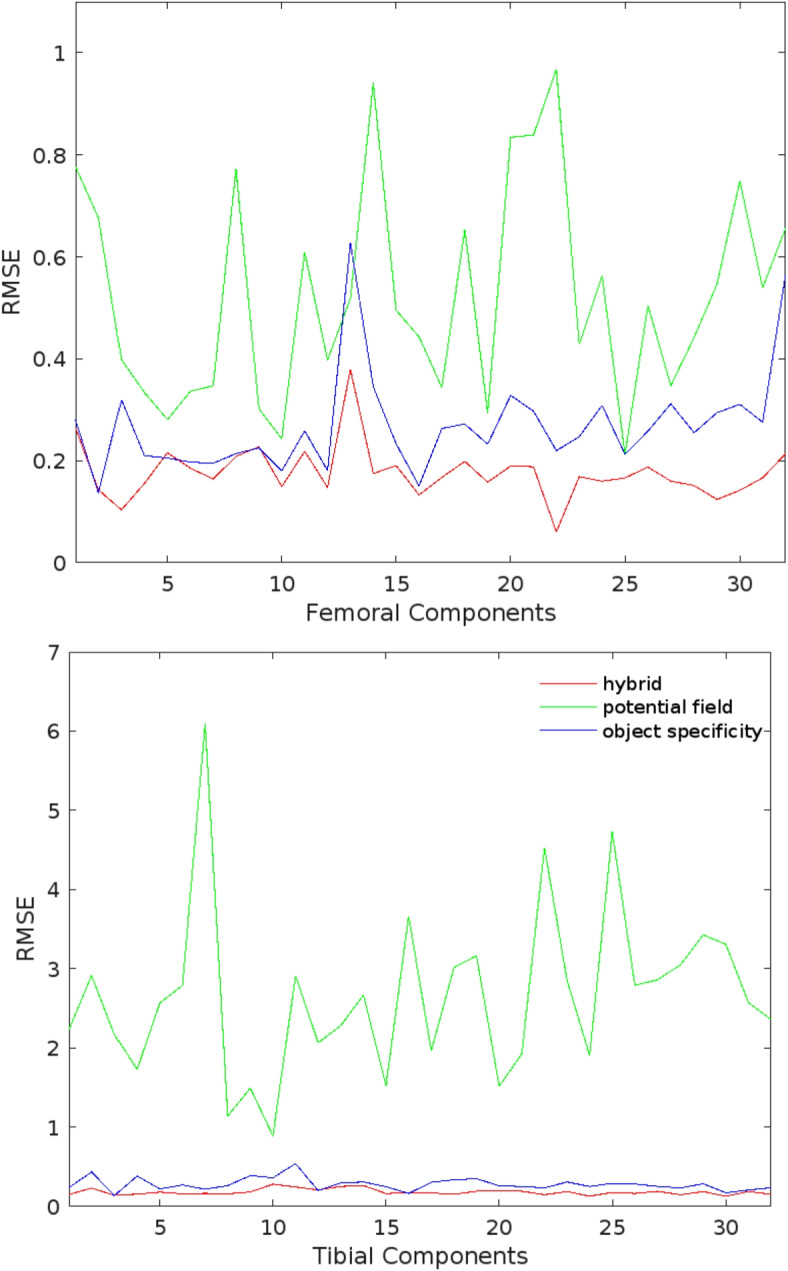
RMSE of each transformed components

**Fig. 7:**
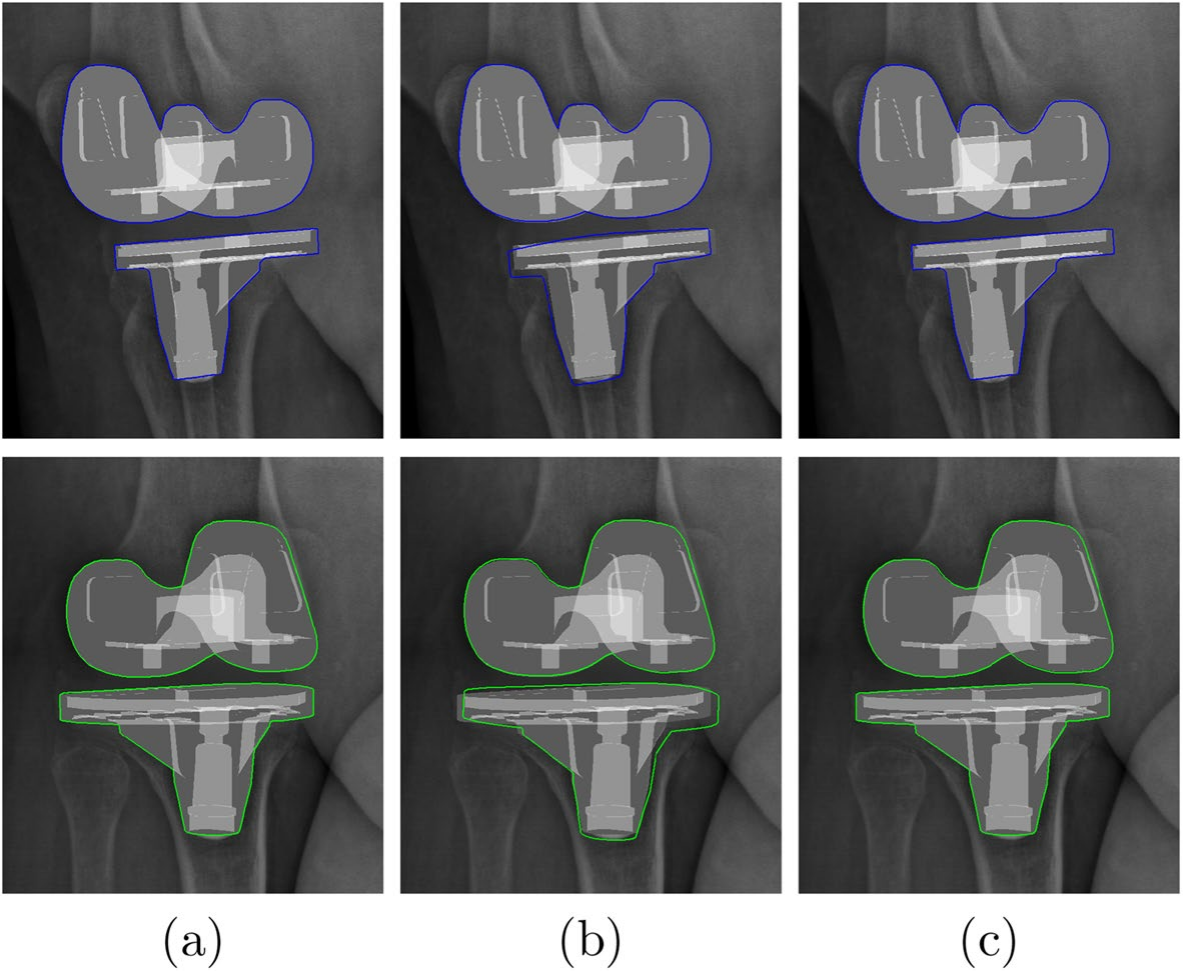
Example of 3D/2D registration result on ground truth images: **a** hybrid similarity, (**b**) potential field similarity, and (**c**) object specificity similarity, first row : 45-degree image and second row : 135-degree image

**Fig. 8:**
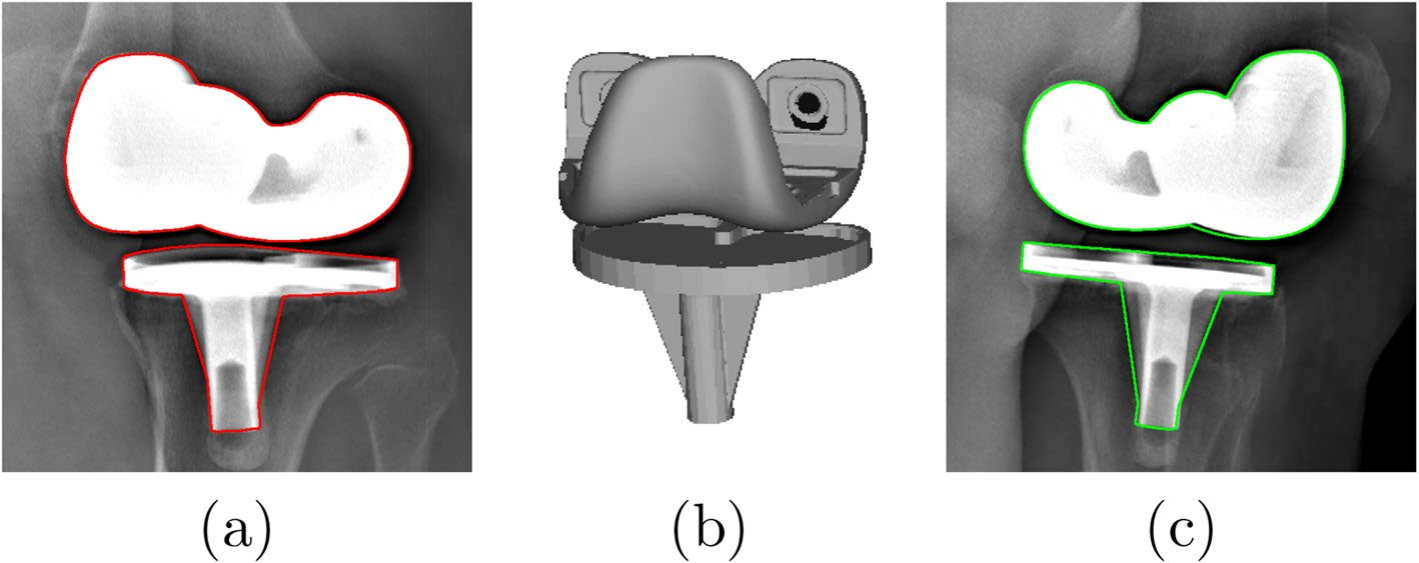
Example of 3D/2D registration result on real radiographic images. **a**, **c** component external contours projected on 45-degree image and 135-degree image, and (**b**) 3D view

Table 1 shows the average RMSE^2^ for the random transformed components before registration. Table 2 shows the average RMSE for each component and for all components by using potential field similarity, object specificity similarity and our hybrid similarity in our accuracy test, as well as the comparison between the different approaches. Table 3 shows the average errors (AE) and the standard deviations (SD) by using single similarities and hybrid similarity, and the comparison between each DOF. Figure 6 shows the RMSE of each transformed components. Finally Figs. 7 and 8 show an example of 3D/2D registration on ground truth images and real radiographic images, respectively.

**Table 1:** Average of RMSE (mm) for the random transformed components before registration

	Initial RMSE
**Femoral components**	12.055
**Tibial components**	11.481
**All components**	11.768

**Table 2:** Accuracy test (average of RMSE (mm) and *p*-value)

	Hybrid	Potential field	Object specificity
**Femoral components**	0.177	0.525	0.269
**Tibial components**	0.183	2.658	0.28
**All components**	0.18	1.592	0.275
***p***-value ${}_{Hybrid/...}$		$<0.0001$	$<0.0001$

**Table 3:** Average errors ± standard deviations and *p*-values of six DOFs

		Rotation (degrees)			Translation (mm)		
		X	Y	Z	X	Y	Z
**Femoral components**	Hybrid	1.047	0.767	1.062	0.046	0.047	0.086
		± 0.771	± 0.591	± 0.672	± 0.03	± 0.031	± 0.043
	Potential field	1.065	0.822	0.879	0.187	0.161	0.142
		± 0.87	± 0.68	± 0.771	± 0.146	± 0.115	± 0.127
	Object specificity	1.02	0.869	0.977	0.075	0.065	0.101
		± 0.767	± 0.674	± 0.722	± 0.049	± 0.052	± 0.07
**Tibial components**	Hybrid	0.727	0.713	0.878	0.085	0.075	0.046
		± 0.567	± 0.473	± 0.578	± 0.046	± 0.039	± 0.035
	Potential field	2.635	4.854	3.241	0.829	0.777	0.561
		± 1.9	± 3.492	± 1.765	± 0.368	± 0.67	± 0.554
	Object specificity	0.641	0.776	0.869	0.073	0.109	0.067
		± 0.523	± 0.595	± 0.61	± 0.062	± 0.068	± 0.05
**All components**	Hybrid	0.887	0.74	0.97	0.065	0.061	0.066
		± 0.695	± 0.536	± 0.634	± 0.044	± 0.038	± 0.044
	Potential field	1.85	2.838	2.06	0.508	0.469	0.351
		± 1.673	± 3.224	± 1.803	± 0.426	± 0.571	± 0.453
	*p*-value$_{Hybrid/PotentialField}$	$<0.0001$	$<0.0001$	$<0.0001$	$<0.0001$	$<0.0001$	$<0.0001$
	Object specificity	0.83	0.822	0.923	0.074	0.087	0.084
		± 0.684	± 0.637	± 0.67	± 0.056	± 0.064	± 0.063
	*p*-value$_{Hybrid/ObjectSpecificity}$	0.5093	0.2225	0.5552	0.0989	$<0.0001$	0.0012

In our experiments, we set the size of the population to 20 and the number of iterations to 800. For the tibial components, the average RMSE were 2.66 mm, 0.28 mm and 0.18 mm by using the edge potential field-based similarity measure, the object specificity similarity measure and our hybrid similarity measure, respectively. The average RMSE of the femoral components in these three similarity measures were 0.53 mm, 0.27 mm and 0.18 mm, respectively. Finally, for all components, the average RMSE were 1.59 mm, 0.28 mm and 0.18 mm, respectively, and the *p*-values between hybrid similarity and single similarity approaches were inferior than 0.0001. A complete evaluation took, on average, approximately 245 seconds on a 64-bit desktop PC (Ubuntu 16.04 LTS, 1.30GHz Core i7 CPU and a graphic card with Intel, 16GB RAM). Note that we didn’t use any libraries in programming in C++.

## Discussion

Our tests showed that the potential field similarity is sensitive. Its average RMSE of the tibial components was a lot higher than those of the femoral components because there are more artifacts around the tibial component than the femoral component. Tibial component contours are attracted by the potential field of the tibial and fibular contours. The object specificity similarity was more stable and accurate than the potential field similarity. But the combination of both edge and region-based similarity measures gave the best result in terms of stability and accuracy, as shown by the statistically significant difference that was found between hybrid similarity and single similarity approaches. The difference of their average RMSE of each component was less than 0.006 mm. The advantage of label-based similarity is its stability. In addition, this term, based on the region process and a numerical integration is inherently robust to noise. However, this measure alone is not precise because the number of pixels in the border labels is fewer than the number of pixels inside the label. On the contrary, the edge-based similarity measure is accurate while also being more sensitive to noise or other artifacts existing in the images. That’s why the combination of the stability and the robustness to noise of the label-based similarity term with the accuracy of the edge-based similarity measure, provides a robust unsupervised registration method.

Compared to the NCC-based method in [25], the experiments showed that their results are very slightly better than that of our method (see Table 4). The differences were less than 0.04 mm. Note that the tests of the NCC-based method used fluoroscopic images of sawbones and didn’t involve the rotations.

**Table 4:** Results (average of RMSE (mm)) of our method versus the NCC-based method in [25]

	Our method	NCC-based method
**Femoral components**	0.177	0.141
**Tibial components**	0.183	0.15
**All components**	0.18	0.146

**Fig. 9:**
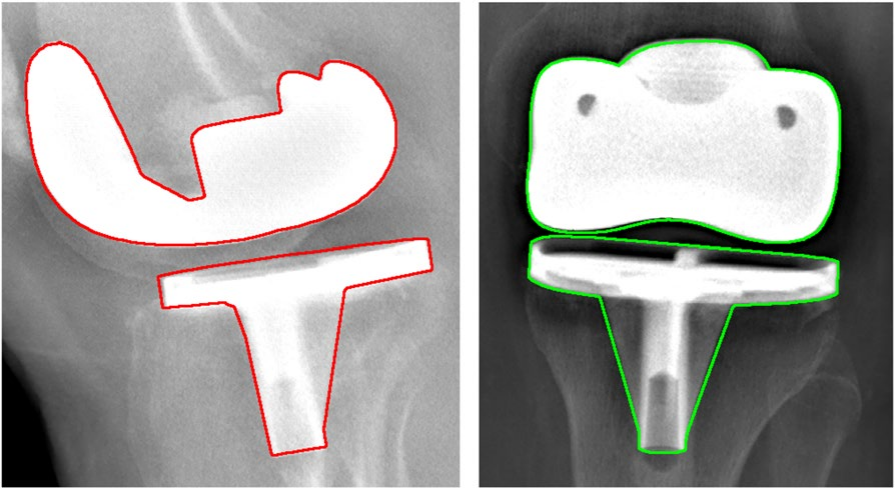
Example of our registration for femoral and tibial components on real radiographic images : lateral image (left) and frontal image (right)

**Fig. 10:**
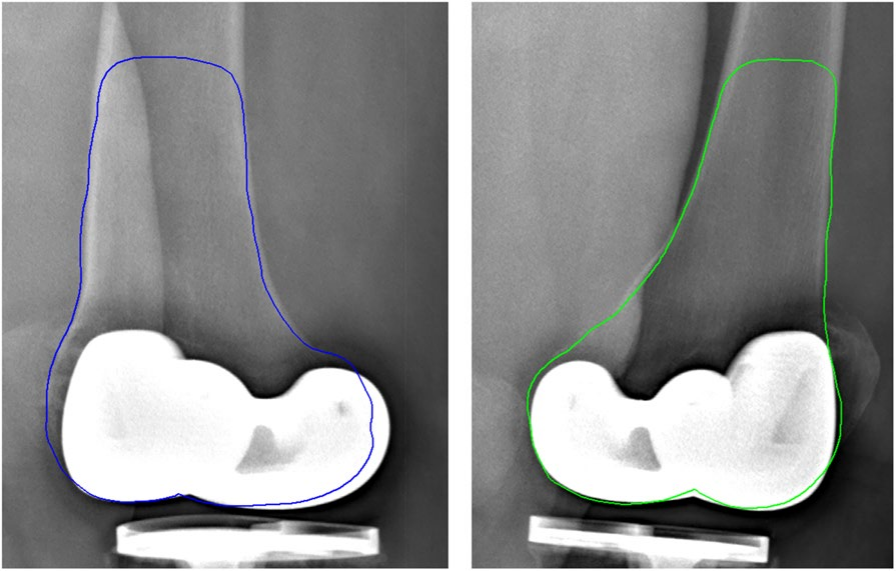
Example of our registration for the distal femur on real radiographic images : 45-degree image (left) and 135-degree image (right)

Unlike other works that use fluoroscopic images, our method uses biplanar radiographic images which are the advantageous in terms of cost, complexity, and risk of radiation and provide six registration parameters with a sufficient accuracy without the need for additional fiducial markers. Our method is also robust to image noise and occlusions, as demonstrated by the AE and the SD of each parameter in our tests. The AE of translation parameters were around 0.06 mm and their SD were less than 0.05 mm. For the rotation parameters, the AE were less than 1 degree, and the SD were around 0.6 degree. In addition, this method can be extended to register other implants or bones to biplanar oblique or frontal/lateral X-ray images (see Figs. 9 and 10).

The proposed method, based on the ES optimizer, is slightly time-consuming but it is easily parallelizable and thus remains especially well-suited for the next-generation GPU or massively parallel computers and multi-core processors.

Based on the result of our TKA component registration, the rotational alignment of the femoral and tibial components can be studied by measuring and analyzing both component position and orientation. For example, the external rotation angles of the implants can be measured. These rotations are important in patello-femoral tracking because inappropriate rotation of the femoral component may cause flexion imbalance and patellofemoral problems [25]. The combined rotational alignment change after TKA can also be measured and the different influence of symmetric and asymmetric tibial component designs on the combined rotational alignment can be compared [36]. Our accurate registration method makes it possible to perform these important analyses on a large number of cases.

## Conclusion

We have presented an unsupervised registration of 3D knee implant components to biplanar X-ray images. This method uses a hybrid similarity measure by combining the object specificity property and the similarity between the external contours of the component projections and an edge potential field (related to the edges) estimated on the two radiographic images. A stochastic optimizer (ES) algorithm is then used to efficiently estimate the six DOFs of implant position. Our method can avoid symmetrical solution and provides six registration parameters with a good accuracy. Moreover, it does not require any fiducial markers. The proposed 3D/2D registration approach has the potential to increase the effectiveness of computer-aided clinical analysis, namely relative angle analysis which is important to predict not only the function but also the stability and survival of TKA implants [37].

## Data Availability

Not applicable.

## Abbreviations

2D: Two-dimensional
3D: Three-dimensional
5D: Five-dimensional
TKA: Total knee arthroplasty
RMSE: Root mean square error
MRI: Magnetic resonance imaging
CT: Computed tomography
RSA: Roentgen stereophotogrammetric analysis
DOF: Degree of freedom
NCC: Normalized correlation coefficient
ES: Exploration selection
LSQ: Least squares
SLIC: Simple linear iterative clustering
AE: Average errors
SD: Standard deviation

## Footnotes

1 Z-score=$\frac{\mu _{2} - \mu _{1}}{\sqrt{\sigma _{1}^2 / n}}$, where $\mu _{2}$ is the average error of the single similarity approach, $\mu _{1}$ and $\sigma _{1}^2$ are the average error and the variance of the hybrid similarity approach, and *n* is the number of components.

2
$RMSE=\sqrt{\frac{\sum _{i=1}^{n} dist_{i}^{2}}{n}}$, where $dist_{i}$ is the distance between a vertex of the transformed component and its correspondence in the ground truth component, and *n* is the number of vertices.
